# Exploring the electrophysiology of Parkinson’s disease with magnetoencephalography and deep brain recordings

**DOI:** 10.1038/s41597-024-03768-1

**Published:** 2024-08-15

**Authors:** Fayed Rassoulou, Alexandra Steina, Christian J. Hartmann, Jan Vesper, Markus Butz, Alfons Schnitzler, Jan Hirschmann

**Affiliations:** 1 Institute of Clinical Neuroscience and Medical Psychology, Medical Faculty, Heinrich Heine University, 40225 Düsseldorf, Germany; 2 Center for Movement Disorders and Neuromodulation, Department of Neurology, Medical Faculty, Heinrich Heine University, 40225 Düsseldorf, Germany; 3 Department of Functional Neurosurgery and Stereotaxy, Medical Faculty, Heinrich Heine University, 40225 Düsseldorf, Germany

**Keywords:** Parkinson's disease, Medical research

## Abstract

Aberrant information processing in the basal ganglia and connected cortical areas are key to many neurological movement disorders such as Parkinson’s disease. Investigating the electrophysiology of this system is difficult in humans because non-invasive methods, such as electroencephalography or magnetoencephalography, have limited sensitivity to deep brain areas. Recordings from electrodes implanted for therapeutic deep brain stimulation, in contrast, provide clear deep brain signals but are not suited for studying cortical activity. Therefore, we combine magnetoencephalography and local field potential recordings from deep brain stimulation electrodes in individuals with Parkinson’s disease. Here, we make these data available, inviting a broader scientific community to explore the dynamics of neural activity in the subthalamic nucleus and its functional connectivity to cortex. The dataset encompasses resting-state recordings, plus two motor tasks: static forearm extension and self-paced repetitive fist clenching. Most patients were recorded both in the medicated and the unmedicated state. Along with the raw data, we provide metadata on channels, events and scripts for pre-processing to help interested researchers get started.

## Background & Summary

Parkinson’s disease (PD) is a neurological movement disorder characterised by degeneration of dopaminergic neurons in the midbrain, leading to impaired information processing in the basal ganglia and associated cortical areas^1^. As both subcortical and cortical signalling is affected, it is important to study the electrophysiology of extended subcortico-cortical networks^2^. While this is feasible in animal models, it is challenging in patients. Non-invasive techniques, such as electroencephalography (EEG) and magnetoencephalography (MEG), struggle to obtain signals from deep brain locations that are critical for understanding the disease. Deep brain stimulation (DBS) electrodes, in contrast, provide direct access to these areas when used for recording rather than stimulation. Yet, they are not suited for studying cortical activity.

**Fig. 1:**
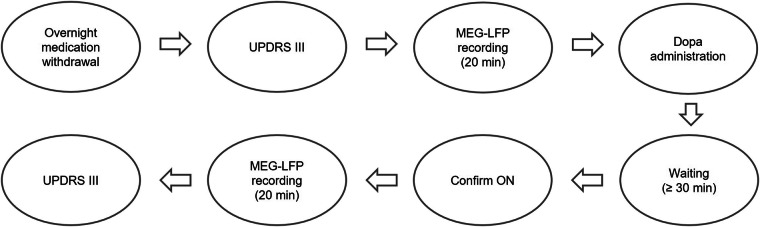
Study design. The measurements were performed the day after electrode implantation using externalized leads. The pulse generator was implanted the day after the measurement.

Here, we provide a dataset allowing for a range of analyses in patients, and the study of subcortico-cortical networks in particular. This dataset contains simultaneous MEG and deep brain recordings of 20 PD patients who were implanted with electrodes for therapeutic deep brain stimulation of the subthalamic nucleus (STN) the day before measurement. We acquired data from most subjects in both the medicated and unmedicated state and quantified motor symptom severity using the Unified Parkinson’s Disease Rating Scale (UPDRS) part III^3^. Figure 1 depicts an overview of the study design.

The combination of MEG and deep brain recordings is rare and available in only a few centers worldwide^4–6^. Being a highly limited resource, such data remain unavailable to most researchers in the field unless they are shared with open access. In doing so, we hope to excel the exploration of neural activity in human basal ganglia-cortex loops.

The data offer several possibilities for sophisticated analyses, including verification and extension of previous analyses. The original aims of the study were to uncover resting-state STN-cortex networks^7^ and to elucidate the modulations of brain activity associated with voluntary hand movement^8^ and tremor^9,10^. The data have further been used in studies exploring the predictive potential of electrophysiological signals with respect to individual symptoms and therapeutic benefit, leveraging machine learning techniques^11–13^. Besides building on these previous analyses, there are countless possibilities to use the data in novel ways. This might include testing and informing computational models of basal ganglia-cortex loops, using the deep brain recordings as a ground truth for MEG source estimates of deep brain activity, or comparisons to similar datasets. To enable these and similar endeavors, we here present a structured and annotated dataset, a comprehensive description of the experimental protocol and of the dataset constituents, a technical validation addressing data quality, and Python code to get interested researchers started.

## Methods

### Participants

20 PD patients eligible for STN DBS (six females), aged between 47 and 75 years (median age: 63 years), participated in the study with written informed consent in accordance to the Declaration of Helsinki (Table 1). Data collection was approved by the Ethics Committee of the Medical Faculty of Heinrich Heine University Düsseldorf (study no. 3209). Participants’ consent to the open publication of the anonymized data was waived by the Data Protection Office of University Clinic Düsseldorf.

**Table 1:** Patient information.

Participant ID	Age [y]	Sex	Hand Moved	Disease Duration [y]	UPDRS OFF	UPDRS ON	Electrode Model
sub-0cGdk9	48	m	l	10	50	32	MDT
sub-2IU8mi	60	m	l	6	43	38	MDT
sub-2IhVOz	69	m	r	11	39	31	MDT
sub-6m9kB5	53	m	—	6	27	21	BSC
sub-8RgPiG	53	m	—	9	24	9	BSC
sub-AB2PeX	64	f	l	14	27	21	MDT
sub-AbzsOg	61	f	l	10	56	32	MDT
sub-BYJoWR	54	f	r	10	47	16	MDT
sub-FIyfdR	70	f	r	4	41	36	MDT
sub-FYbcap	66	m	l	8	40	29	MDT
sub-PuPVlx	71	m	l	6	51	17	MDT
sub-QZTsn6	72	m	l	17	36	—	MDT
sub-VopvKx	70	m	r	11	39	21	MDT
sub-dCsWjQ	68	m	l	6	39	20	MDT
sub-gNX5yb	62	f	l	15	31	20	MDT
sub-hnetKS	76	f	l	21	34	13	MDT
sub-i4oK0F	69	m	l	1	18	4	MDT
sub-iDpl28	53	m	r & l	11	55	33	MDT
sub-jyC0j3	62	m	r	16	26	13	MDT
sub-oLNpHd	54	m	l	12	27	—	SJM
**Mean**	**62.7**			**10.2**	**37.5**	**22.5**	
**Median**	**63**			**10**	**39**	**21**	
**SD**	**8**			**4.7**	**10.5**	**9.5**	

Age, disease duration and motor symptom severity (Unified Parkinson’s Disease Rating Scale Part III) refer to the date of measurement. MDT: electrode with 4 ring contacts by Medtronic (model 3389); ø1.27 mm; contact length: 1.5 mm; contact spacing: 0.5 mm; BSC: electrode with 8 ring contacts by Boston Scientific (model Vercise Standard); ø1.3 mm; contact length: 1.5 mm; contact spacing: 0.5 mm; SJM: electrode with 4 ring contacts by St. Jude Medical (model 6148); ø1.4 mm; contact length: 1.5 mm; contact spacing: 0.5 mm. m = male, f = female, l = left, r = right. OFF: without medication. ON: with medication.

### MEG System and recording setup

All recordings were conducted using a 306-channel whole-head MEG system (VectorView, MEGIN). Simultaneously, local field potentials (LFPs) from the subthalamic nucleus were recorded. The recording sessions took place the day following electrode implantation. In this phase, the stimulator had not been implanted yet. All patients were implanted with standard DBS electrodes (Table 1), having either 4 or 8 ring contacts. The electrodes were externalized, i.e. equipped with extension cables that allowed us to connect the electrodes to the built-in amplifiers of our MEG system. To ensure minimal magnetic interference, we used custom-made, non-ferromagnetic extensions (Medtronic Bakken Research Center, Maastricht, the Netherlands).

### Acquisition

During the recording sessions, we simultaneously acquired various types of electrophysiological data, including MEG, LFPs, vertical and horizontal electro-oculograms, and the EMG of the extensor and flexor muscles of both forearms. The DBS electrode contacts were referenced against a surface electrode located at the left mastoid. EMG signals were recorded with reference to surface electrodes attached to the forearm tendons. A sampling rate of 2 kHz was used to capture the data. Online band-pass filtering was applied to MEG signals within the range of 0.03 to 660 Hz, while LFP and EMG signals were filtered between 0.1 and 660 Hz.

### Medication withdrawal and OFF state verification

On the evening before the recordings, dopaminergic medication was discontinued until the end of the medication OFF measurement next morning. In case patients were equipped with an apomorphine pump, the pump was stopped at least 2 hours before the recordings started. The medication OFF state was verified by a UPDRS III rating before the recordings started.

### Experimental design

The experiment consisted of four blocks: (i) rest followed by static forearm extension (HOLD) in medication OFF, (ii) rest followed by repetitive fist-clenching (MOVE) in medication OFF, (iii) rest followed by HOLD in medication ON and (iv) rest followed by MOVE medication ON. In patients 8RgPiG and 6m9kB5 we only acquired resting-state data.

In between the OFF and ON blocks, patients received 1.5 times their usual levodopa morning dose (mean = 205 mg, STD = 40 mg, min = 140 mg, max = 300 mg) and we waited for at least 30 minutes for the drug to take effect. Subsequently, we started to re-score selected UPDRS items such as diadochokinesia and finger tapping every ten minutes and continued with the MEG measurement as soon as we observed a clear motor improvement. After the medication ON measurement, we acquired another full UPDRS III score.

In the HOLD condition, patients elevated the forearm of their more affected body side (elbow joint about 60°) and kept this position until instructed via intercom to take a break. This process was repeated several times until we had collected about 300 seconds of forearm extension (M = 309.54, SD = 50.40).

**Fig. 2:**
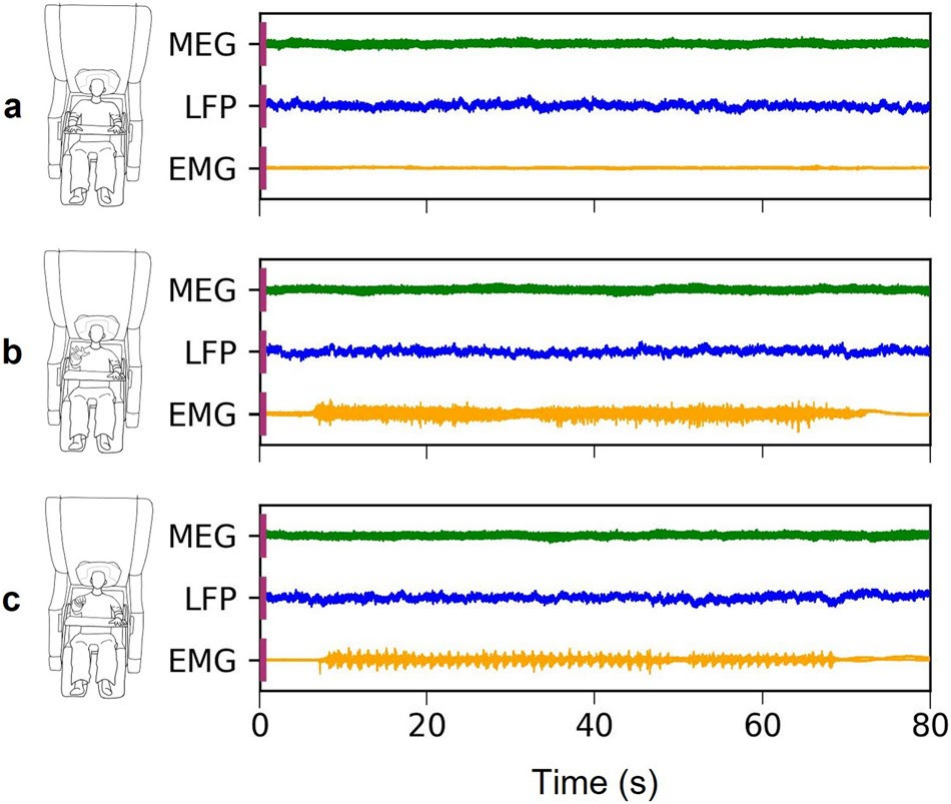
Example data excerpt for each condition. The plots depict one MEG sensor (*green*), one LFP channel (*blue*)*, and* one EMG channel (*yellow*): (**a**) Resting state; (**b**) Static forearm extension; (**c**) Repetitive fist-clenching. Movements are initiated at about 10 s and are terminated at about 70 s.

In the MOVE condition, patients performed repetitive fist-clenching with their more affected hand. Movement was self-paced, but patients were instructed to clench their fist about once per second for 300 seconds (M = 284.62, SD = 44.29). In line with the HOLD condition, we introduced pauses to avoid fatigue. Note that the change of hand position is captured by the EMG (Fig. 2).

### Construction of head and source models

In this dataset, we provide head models and Montreal Neurological Institute (MNI)-aligned source models along with the raw data so that interested researchers can perform source analysis. Realistic, single-shell head model creation for MEG source analysis and the construction of source models were performed using the FieldTrip toolbox in MATLAB R2020a. The head models are based on individual T1-weighted MR images of the patients, which we segmented into different tissue types using SPM routines via the function *ft_volumesegment*. The triangulated brain-skull boundary served as the single shell, surface head model. Relating the head model to the MEG sensors in space (MEG-MRI co-registration) was achieved by identifying three anatomical landmarks, namely nasion, right and left periauricular point, in both the MRI and the MEG coordinate system. The former involved visual inspection of the MRI and marking the landmarks by hand. The latter was achieved by first determining the position of each landmark relative to four magnetic coils, attached to the subjects’ head, using a Polhemus digitizer system. The coils, capable of producing a small current, were source-localized in the head position indicator (HPI) measurement preceding each MEG recording. Once the landmark coordinates are known in both coordinate systems, the MRI/head model and the sensors can be represented in the same coordinate system (defined by the landmarks). The forward solution was approximated using the theorem for the magnetic lead field in the quasi-static approximation^14^.

The MNI-aligned source models, henceforth referred to as grids, define a set of locations spanning the entire brain. 3D coordinates in individual head space can be found in the grid.pos field (N × 3 matrix, with N being the number of locations). During source reconstruction, brain activity is estimated for each of these locations. The grids were constructed by first normalizing the individual MRI, using SPM routines via *ft_volumenormalise*. The inverse of the resulting nonlinear transformation was then applied to a regular MNI template grid with a spacing of 4 mm. After the transformation, the template grid fits the individual MRI, but the regular spacing is lost. Importantly, each position in the MNI-aligned grid has a corresponding position in the template grid (X_ind_,Y_ind_,Z_ind_ in row *i* of individual_grid.pos corresponds to X_MNI_,Y_MNI_,Z_MNI_ in row *i* of template_grid.pos). When using these grids, one effectively estimates brain activity in homologous locations in each subject. The MNI coordinates of these locations are known and can be used to look up anatomical labels in an atlas, for example, or to plot source activity in a standard brain. The latter is particularly useful for group analyses of source images. These steps are exemplified in the Matlab script  *source_analysis_example*  available on GitHub (https://github.com/Fayed-Rsl/RHM_preprocessing).

### Technical validation methods

The recordings were visually inspected and segments with artifacts were discarded (1.79% of the data on average; SD = 2.51, range: 0 to 11%). For technical validation, we re-referenced the LFPs using a bipolar referencing scheme by subtracting signals from neighbouring electrode contacts within each hemisphere in order to focus on local neural activity^15^.

Minimal pre-processing included a 1 Hz high-pass filter to remove low-frequency noise and slow drift, as well as down-sampling the data from 2000 to 200 Hz. The power spectral density of the EMG signals from the hand performing the task was computed using Welch’s method in the frequency range from 2 to 45 Hz^16^. For computing resting-state MEG-LFP coupling, we created fixed-length epochs lasting 2-s with a 1-s overlap. The epochs were convolved with 7 Slepian tapers, and coherence between MEG and LFP was computed using the *spectral_connectivity_epochs* function of the MNE Python toolbox in the frequency range from 13 to 30 Hz^17^.

Electrode placement was assessed using LeadDBS, a Matlab toolbox for reconstructing the location of DBS electrodes from their characteristic CT artifacts and visualizing them in a common template brain^18^. This step required an individual pre-surgical MR and a post-surgical CT scan.

## Data Records

**Fig. 3:**
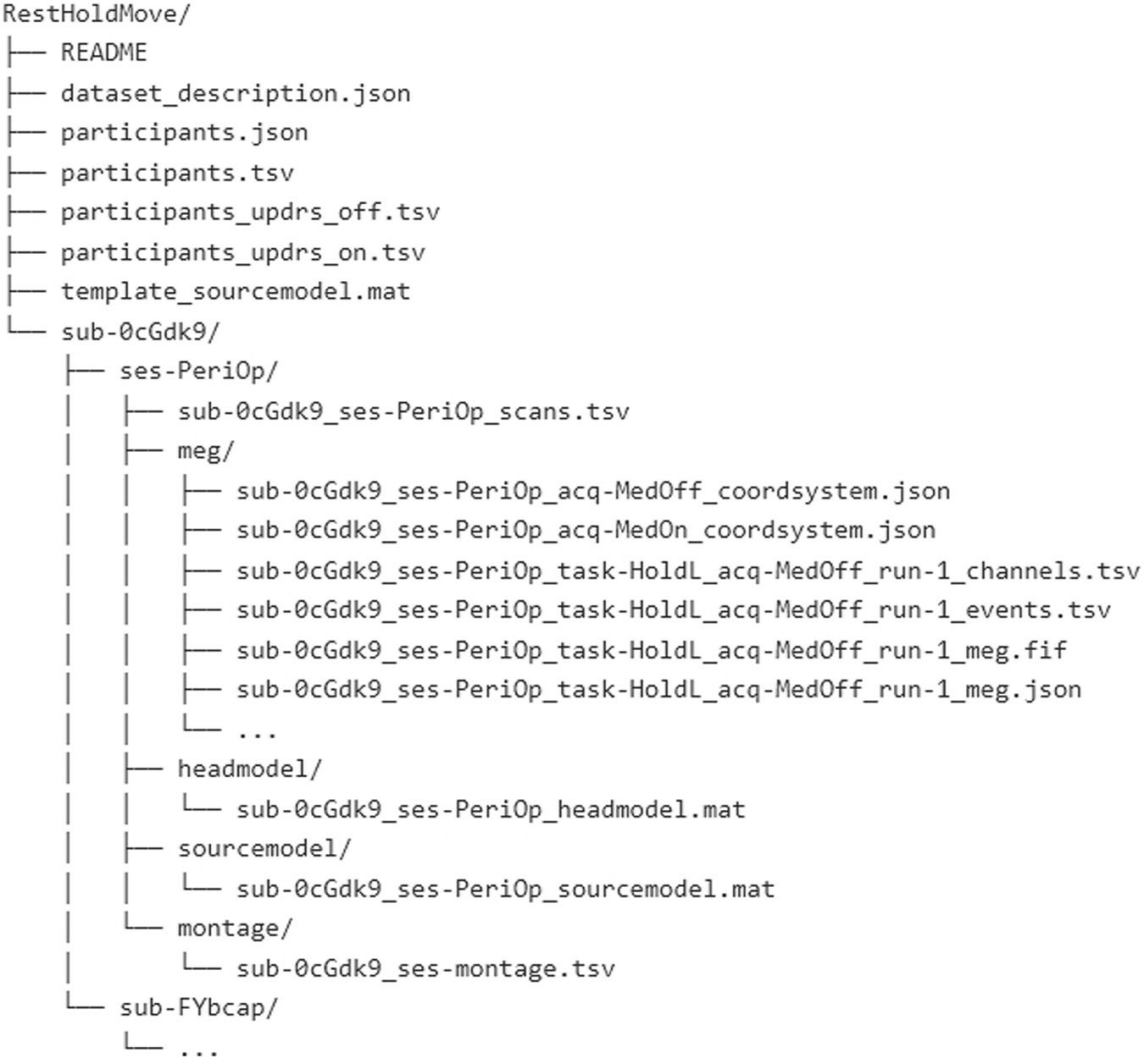
Dataset structure. Brain Imaging Data Structure (BIDS) showcasing the hierarchical arrangement of files.

The dataset adheres to the Brain Imaging Data Structure (BIDS) v1.6.0 standard and was transformed into BIDS format using the MNE-BIDS tool (https://github.com/mne-tools/mne-bids) version 0.10^19,20^. It is publicly accessible at https://openneuro.org/datasets/ds004998^21^ and has a total size of 162GB. The dataset’s structure is outlined in Fig. 3.

The directory structure consists of a root folder containing general meta-information and the individual subject folders (e.g., sub-0cGdk9). Each subject-folder contains one session-folder called *ses-PeriOp* with the raw data and session-specific meta-information. All electrophysiological recordings are contained in the.fif files (MEG, LFP, EOG and EMG). Rest and motor task (either HOLD or MOVE) are part of the same file, unless the patient had only undergone resting-state recordings. Each.fif file has a size of approx. 1.5GB, comprising 204 gradiometers, 102 magnetometers, 8 LFP channels (prefix “EEG”), 4 EMG channels, 2 EOG channels and 3 “stimulus channels”. The latter can be used to record external triggers, but were not used here.

In order to epoch the data into rest, forearm extension, and fist-clenching, we provide both the epoch start and end times <*events.tsv*> and code for epoching (https://github.com/Fayed-Rsl/RHM_preprocessing). In addition, we provide a list of bad channels <*channels.tsv*> and epochs containing artifacts <*events.tsv*>, which should be removed in pre-preprocessing. Typical reasons for marking a channel/epoch as bad are SQUID jumps (MEG) or strong high-frequency noise caused by muscle contractions. The mapping from LFP channel labels, as contained in the raw data, to interpretable DBS electrode contact names is provided in <*montage.tsv*>. This is useful for re-referencing the LFP data, which is commonly done to focus on local voltage changes.

### Overview of root folder

- *./dataset_description.json* contains information about the dataset, including title, authors, and study details.
- *./participants.tsv* stores subject identifiers, age, sex, disease and disease duration.
- ./participants_updrs_<on/off>.tsv provides raw, non-aggregated UPDRS scores for each item of part III.
- ./template_sourcemodel.mat contains the template grid (source locations).

### Overview of session folder

- **<***ses>/meg/* encompasses the raw data, including sensor and anatomical landmark coordinates, channel information and event details.
- <*ses>/headmodel/* contains the head model (.mat).
- <*ses>/sourcemodel/* contains the individual MNI-aligned grid (.mat).
- <*ses>/montage/* holds montage-related information for re-referencing LFP data.

## Technical Validation

**Fig. 4:**
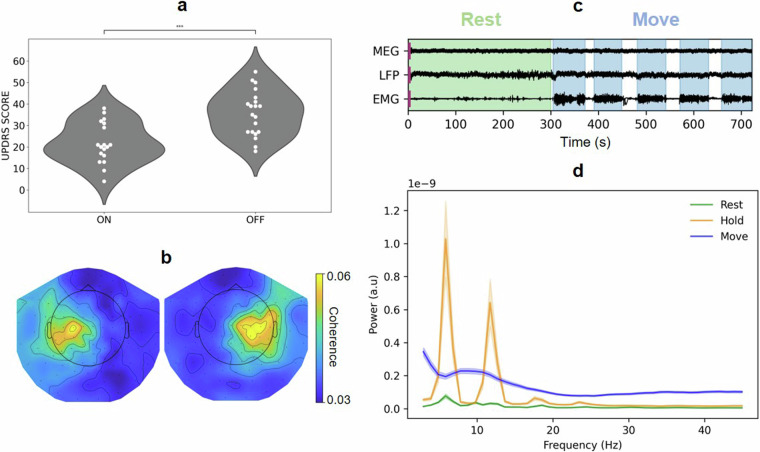
Technical validation. (**a**) Unified Parkinson’s Disease Rating Scale (UPDRS) Part III scores in medication ON and medication OFF. ***p < 0.01, paired *t-*test. (**b**) Topographical maps of MEG-LFP beta-band coherence, averaged over all left-hemispheric and all right-hemispheric electrode contacts, respectively. (**c**) Example of MEG, LFP, and EMG time-domain data (subject QZTsn6), REST (green), and MOVE (blue) highlighted. (**d**) Group-average power spectral density of EMG signals for REST (green), HOLD (orange), and MOVE (blue).

We used the BIDS-Validator to ensure that the dataset conforms to the standardised brain imaging data structure (https://github.com/bids-standard/bids-validator). To further validate the technical quality of the dataset, we verified that the medication indeed improved motor symptoms. As expected, administration of levodopa led to a significant reduction in symptom scores in the medication ON state compared to the OFF state (*p* < 0.01, paired *t*-test, Fig. 4a).

Furthermore, we confirmed that the event times we provide correctly reflect the patients’ motor state. To do so, we epoched the data based on the events provided and plotted the forearm EMG both in the time (Fig. 4c) and in the frequency domain (Fig. 4d), revealing distinct patterns corresponding to the different movements. As expected, REST exhibited the lowest EMG power. The spectral peaks around 5 Hz and its harmonics are due to Parkinsonian rest tremor. HOLD was characterized by intermediate power levels with prominent peaks reflecting postural tremor. MOVE had the highest power overall and lacked discernible tremor peaks in the group average.

In addition, we verified that resting-state functional connectivity between STN and cortex displays the hemispheric laterality described by several groups^4,22,23^. Specifically, the left STN is expected to couple to the left motor cortex in the beta band (13–35 Hz) while the right STN is connected to the right motor cortex. Figure 4b illustrates the topographical organization of beta-band coherence averaged over all left-hemispheric electrode contacts and all right-hemispheric electrode contacts, respectively. Coupling was clearly lateralized.

**Fig. 5:**
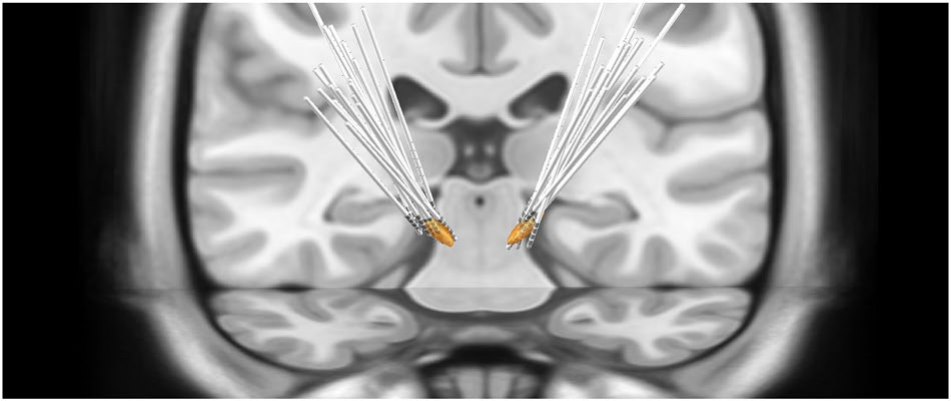
Electrode reconstructions in Montreal Neurological Institute Space. The subthalamic nucleus is shown in orange.

Figure 5 illustrates the reconstructed locations of all electrodes in relation to the STN in MNI space. All electrodes in this dataset appear to be correctly placed. Nonetheless, there is inter-subject variability with respect to contact location.

## Usage Notes

The data is available under a CC0 license and can be retrieved with open access from openneuro.org (https://openneuro.org/datasets/ds004998). To help interested researchers get started with analyzing these data, we provide Python code for loading, epoch creation, artifact rejection and LFP re-referencing on our GitHub repository (https://github.com/Fayed-Rsl/RHM_preprocessing). In this code, we make use of the MNE Python toolbox for MEG data analysis^17^. Note that there are several alternatives, which are equally well suited for this dataset, such as FieldTrip^24^, Brainstorm^25^, or SPM^26^. We additionally provide examples for source reconstruction, based on the FieldTrip toolbox.

For those who have worked with MEG data of healthy participants before, we note that the data quality might be worse than what they are used to. Our data were recorded one day after brain surgery in patients severely affected by PD, off their usual medication. Movement disorders patients cannot be expected to minimize movement the way healthy participants can, resulting in more movement-related artifacts. Rest tremor and postural tremor occurred frequently, for example. The head position was measured at the start of each recording, not continuously.

**Fig. 6:**
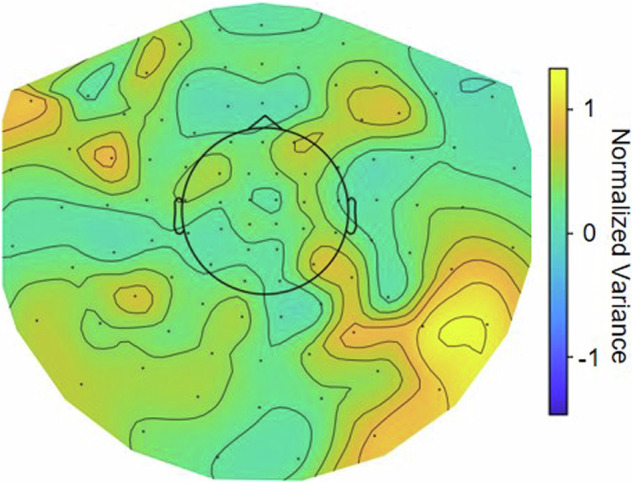
Map of signal distortions caused by electrode extensions. The topographical map represents the group average normalized variance of MEG sensors. We removed sensors with strong noise (>2 SD) and divided each individual variance topography by its mean before averaging over subjects.

Moreover, these patients had brain implants, further affecting signal quality. While the custom-made, non-ferromagnetic extensions used here mitigated most of the severe artifacts related to DBS hardware^27^, the connections were not completely free of ferromagnetic material. This resulted in signal distortions in right parietal and right temporal MEG sensors, reflecting the path of the extensions (Fig. 6). These rather mild artifacts can be dealt with by standard analysis steps such as temporal signal space separation^28^, computing condition contrasts or relating the MEG signal to a non-distorted reference, e.g. by computing LFP-MEG coherence. Please note that artifacts also occur in the LFP data, and noisy LFP channels are common. Hence, screening for artifacts and the removal of bad channels is essential when analysing these data. We provide information and code to assist with this.

Further, we strongly recommend re-referencing the LFP signal, e.g. by subtracting the signals from adjacent contacts (the shared data are nor re-referenced yet). Otherwise, the LFP signal is dominated by muscle activity recorded at the mastoid reference. In this context, please note that the LFP channel labels in the raw data (EEG001, EEG002, …) are not meaningful, but need to be renamed as indicated in the montage.tsv files. The channel labels in these files contain information about the position of each contact along the electrode. LFP-left-0, for example, would indicate the lowermost contact of the electrode in the left hemisphere. Note, however, that we do not provide information about the anatomical position of the contacts, e.g. with respect to the boundaries of the STN or compartments of the STN, due to uncertainty in the electrode reconstruction and the uncertain spatial coverage of LFP recordings. In case an analysis requires contact selection, we recommend a functional criterion such as a threshold on task- or symptom-related activity.

Lastly, we note that the time we waited to reach the OFF state is insufficient for the drugs to lose their effects completely^29^. Thus, the medication OFF state is to be understood as a relative OFF which can be further attenuated due to the surgery-related stun effect.

## Data Availability

All custom code used for pre-processing and technical validation is available at https://github.com/Fayed-Rsl/RHM_preprocessing.
